# Experiences of Assyrian refugee women seeking care for chronic pain: a qualitative study

**DOI:** 10.1186/s12939-023-01891-w

**Published:** 2023-05-08

**Authors:** Areni Altun, Helen Brown, Elizabeth Sturgiss, Grant Russell

**Affiliations:** 1 Department of General Practice, Monash University, 553 St Kilda Road, Melbourne, VIC 3004 Australia; 2 Deakin University, Burwood, VIC Australia; 3 Academic Unit of General Practice, Australian National University, Canberra, Australia

**Keywords:** Primary care, Chronic pain, Qualitative, Refugee women, Care experience

## Abstract

**Background:**

Refugee women exhibit some of the highest rates of chronic pain yet the diversity and challenges of health care systems across countries pose numerous challenges for refugee women trying to access quality health care.

**Objective:**

We sought to explore the experiences of Assyrian refugee women seeking care for chronic pain.

**Methods:**

Semi-structured interviews (face-to-face and virtual) were undertaken with 10 Assyrian women of refugee background living in Melbourne, Australia. Audio recordings and field notes of interviews were collected and themes were identified using a phenomenological approach. Women were required to be conversant in English or Arabic and willing to use a translator if necessary.

**Results:**

We identified five major themes of women’s experiences accessing care for chronic pain: (1) the story of pain; (2) the experience of help seeking in Australia and home country; (3) factors shaping the ability to access appropriate care; (4) support seeking systems; and (5) influence of culture and gender roles.

**Conclusion:**

Exploring refugee women’s experience of seeking care for chronic pain reinforces the need to explore hard to reach population’s perspectives in research and helps to understand how vectors of disadvantage may intersect. For successful integration into health care systems of host countries, particularly for complex conditions such as chronic pain, there is a need to work with women community members to develop programs that are culturally aligned to enhance access pathways to care.

**Supplementary Information:**

The online version contains supplementary material available at 10.1186/s12939-023-01891-w.

## Background

An estimated 1 in 5 adults live with chronic pain worldwide [1]. Chronic pain, defined as pain that lasts longer than three months, or in many instances beyond its normal tissue healing time frame [2], presents a major public health challenge with widespread and serious debilitating effects on individuals [2]. Whilst chronic pain is typically poorly recognised, under-estimated and inadequately managed in the general population [3, 4], the burden of chronic pain is far greater for those who face cultural, financial or geographical barriers to health care [5]. Populations that are systematically disadvantaged are also more likely to live with chronic pain, and with greater pain severity [5, 6]. Adequate management of chronic pain often requires a multidisciplinary approach; however, individuals who have been excluded from societal power structures and decision-making processes face additional challenges when accessing resources and opportunities for effective chronic pain care [7]. This is further compounded by perceived stigmas related to race, ethnicity and gender and may explain why chronic pain is recognised as a defining feature among the wide range of health-related problems exhibited in refugee women [8].

Although access to healthcare is a fundamental human right, the dimensions and complexities of health care accessibility are dynamic and exist in both health systems and population contexts [9, 10]. Seeking care is often associated with stigma and discrimination and becomes more important to consider with population groups that overlap with several sociocultural characteristics [11]. There are also socio-cultural power dynamics that cause many individuals, particularly refugee women, to suffer from subordination leading them to have unequal access to decision making resources such as healthcare [10, 12, 13]. Health care for people of refugee background in nations of resettlement is difficult, costly and often of varying quality. These challenges are magnified when diversity of backgrounds intersect with more complex health needs, poor English language skills and limited health system literacy [13].

The pre and post migration stressors associated with forced displacement and migration can lead to complex mental and physical health issues for women of refugee background [14]. This hardship, trauma and discrimination may often continue as they face uncertainties of their future and socio-economic circumstances upon resettlement. Women’s needs and expectations are qualitatively different from men [15, 16] and forced displacement can create an environment that may diminish their own agency around healthcare decisions and lead them to place their own needs behind those of their family. It is therefore important to ensure women of refugee background are able to engage effectively with primary health care to address their health care needs [14].

Equitable access to primary care is an integral component of chronic pain management and strong primary care systems in many countries are associated with improved health outcomes. Primary care, often considered first-contact care is well positioned to offer person-focused, population-based care, and can facilitate comprehensive coordinated care for complex health conditions such as chronic pain with other referral-based networks. However, with global civil unrest on the rise, primary care services in host nations such as Australia may see greater numbers of refugee and migrant patients with complex health needs and it is unclear whether people of refugee and migrant background are able to access these services based on their needs. In order to provide effective and culturally sensitive care, health services must better understand the changing population to tailor health service delivery to meet the differing needs of people from refugee-like backgrounds [17–19].

Considering these challenges and the diversity of healthcare systems across countries, there is a need to understand the access experiences of populations that are systematically marginalised. Chronic pain management in people of refugee background is complex and in times of civil conflict, women’s health and access options disproportionately suffer [7]. The pre and post settlement experiences for refugee women are fraught with adversities and many of these migration experiences have been linked to chronic pain [11]. There are also contextual elements to consider across different cultures. Studying individual ethnic groups may offer some insight on the factors that shape the access experiences of care and may encourage health services to adapt their service delivery to meet the community-specific needs of diverse populations.

One particular ethnic minority living in Australia that faces vulnerability are the Assyrian Iraqi refugees. The majority of Assyrian people were living as Christian minority groups in an otherwise predominant Islamic state and who have now migrated to Australia, facing a very different cultural context [20]. This Iraqi-born population is the eighteenth largest migrant community in Australia, equivalent to 1.2 per cent of Australia’s overseas-born population [21].

Despite having sought refuge in Australia, access to appropriate chronic pain care for people of refugee background, particularly women remains a challenge. To better understand the levers that influence access to chronic pain management our study sought to explore the lived experience of Assyrian refugee women seeking care for chronic pain and understand the factors that influence their decision to seek care for chronic pain in Australia. Insights into the health and socio-cultural experiences of refugee women seeking care for chronic pain may help identify hidden needs and allow for greater understanding of how health systems can address health inequalities exhibited in other minority groups who are systematically marginalised.

## Methods

### Study design

A qualitative methodology was used to explore the lived experience of Assyrian refugee women seeking care for chronic pain. The study focused on the women’s lived experiences and as such, a phenomenological approach was taken [22, 23]. The study was predominantly set in the South East region of Victoria, Australia where a significant proportion of Australia’s refugees are resettled [24]. The study was approved by the Monash University Human Research Ethics Committee in February 2020.

### Participants and recruitment

Purposive sampling with a snowballing strategy was initially undertaken to recruit approximately ten Iraqi refugee women who had lived with or were living with chronic pain (over 3 months) in Australia. Eligible participants were eighteen years and older, who were residing in Melbourne and had entered Australia through a humanitarian refugee visa. These women were required to be conversant in English or Arabic and willing to use a translator if necessary.

Participant recruitment brochures were circulated throughout the Assyrian refugee community with the help of Arabic speaking community leaders working in refugee health services. Assyrian community leaders were identified through pre-existing relationships with settlement services to liaise with potential participants. Potential participants were invited to contact a researcher (AA) directly or were asked to provide permission to be contacted via telephone or email.

AA then contacted potential participants by phone to further explain the study and gain verbal consent, reminding them that participation was voluntary, and they could withdraw from the interview at any time if they chose to do so. Participants who were not able to speak English were contacted by an interpreter to explain the study details and participant information forms were also translated into Arabic for participants not literate in English. All participants were informed that they would receive a small honorarium of $50 AUD (grocery voucher) as compensation for their time.

#### Data collection

The study sought to explore women’s health seeking narratives through semi-structured, in-depth interviews. The interview guide (see Supplementary Material) was informed by an explanatory model of illness developed by Zander et al. 2015 [25]. The interview guide was refined during the study while keeping the overall style conversational and situational. The interview guide incorporated flexibility regarding the order in which the questions were asked, and clarification questions were used when needed. Interviews concluded once a full and complete understanding of the research topic was achieved and data saturation had been met [26]. Field notes were also used to contextualise the interviews and brought into focus deeper meaning and understanding of the culture and social situations.

Data were collected between June and November 2021. A bilingual Arabic speaker, hired through a private organisation and who was of Iraqi origin was available to translate for participants who were not conversant in English. Of the four participants who were not conversant in English, two participants requested to use family members as translators. Interviews were conducted in a location chosen by the participant, usually their home or a local university office, public library or online via zoom. The interviews lasted between 45 and 90 minutes.

The following translation decisions were made: certain phrases and nuances in Arabic expression were discussed with the key informants such as members of the Assyrian community; the researcher transcribed pauses, overlaps, and fragmentary phrases in interviews; and lexical decisions in translation were literal rather than dynamically equivalent [27].

#### Data management

Interviews were audiotaped and field notes of activities, events and interactions were kept. On completion of interviews, de-identified audio recordings were transcribed verbatim in English into a word document. Transcripts were imported into qualitative analysis software NVivo version 12 to help organise the data.

### Data analysis

The research team consisted of AA, a PhD student with professional experience in Osteopathy and three senior qualitative researchers (HB, GR and ES), two of whom are academic family physicians/General practitioners (GPs) (GR and ES). This was important to offer clinical insights into the health-related topics discussed by the participants. A phenomenological approach was used to explore the complex and nuanced accounts of the women’s stories. Initial read through and coding was done separately. Then, the team met regularly to discuss analyses, and categories and sub-category codes. The codes evolved as the analysis progressed with reflexive interpretations of the data with the use of inductive coding early in the analysis process [28]. Following the analysis of codes, themes based on the pattern of shared meaning were developed, united by a central concept for each theme. The themes underwent several revisions by the research team to ensure a correct representation of the concept was being conveyed, using field notes to contextualise the data. Ambiguities were resolved and themes were developed from categories through discussion among the research group members and re-reading of transcripts.

### Reflexivity

Intersecting relationships between participants and researchers play a role in the collection and analysis of qualitative data. All researchers in this study have prior professional experiences working with refugee and/or migrant health issues. Three of the researchers have clinical experience (AA, GR, ES), two are family physicians (GR, ES) and AA is an Osteopath. Hence, to avoid the influence of any pre-conceived assumptions, subjectivities and/or potential prejudices; self-reflexivity was a particularly important component throughout the research process. This was also achieved more broadly through regular research team meetings throughout the data collection and analysis process where possible influences and potential biases were discussed. It was also valuable to have the primary researcher (AA) who is a woman and born to migrant parents (born in Lebanon and Turkey), to be involved in data collection and analysis. Sharing some cultural similarities facilitated comfort and a sense of understanding between the primary researcher (AA) and the participants. This helped to build rapport and created an environment of openness to discuss the intimate details surrounding the many topics of trauma, mental health and their experience seeking care for chronic pain both in Australia and home country.

## Results

### Data and findings

#### Participant characteristics

A total of 10 women aged between 19 and 85 years who had lived in Australia between four to 25 years participated in the study.

Iraq was the country of origin for all participants; however, most had spent between one to three years living in a transit country such as Jordan, before arriving to Australia. Many of these women fled their country bearing witness to war and violence in their home country and are left living in the emotional aftermath of a traumatic event.

Five of the women were married with children, three were widowed and two were not married. Two of the women were currently employed and half had a tertiary degree completed in their nation of origin. All women who reported having chronic pain also reported a comorbid chronic disease such as arthritis, depression, heart disease etc. Demographic characteristics of the study participants are outlined in Table 1.

**Table 1:** Characteristics of Assyrian refugee women living with chronic pain

Participant	Age bracket	Time residing in Australia	Highest level of education	Relationship status	Children	Comorbid disease
P1	18–29	20–25 years	University	Married	2	Cholelithiasis, Bariatric–metabolic surgery
P2	50–60	0–5 years	University	Widow	2	Depression, Anxiety, Osteoarthritis, Eyelid disorder, Thyroid dysfunction
P3	40–50	0–5 years	University	Married	3	Chronic fatigue, Depression, Dysmenorrhea
P4	18–29	0–5 years	TAFE	Not married	0	Obesity, Hypothyroidism, PTSD, Vocal cord dysfunction
P5	40–50	0–5 years	High school	Married	1	Type 2 Diabetes, Breast Cancer, Depression
P6	30–40	15–20 years	High school	Married	5	Insomnia, Depression
P7	18–29	20–25 years	High school	Married	3	Chronic urinary tract infection
P8	80–90	10–15 years	University	Widow	3	PTSD, Depression, Hypertension, Type 2 Diabetes, Hypercholesteremia
P9	50–60	10–15 years	High school	Widow	0	Hypertension, Type 2 Diabetes, Hypercholesteremia
P10	70–80	0–5 years	High school	Not married	0	Hypertension

Five themes were identified. These were (1) the story of pain; (2) the experience of help seeking in Australia and home country; (3) factors shaping the ability to access appropriate care; (4) support seeking systems; and (5) influence of culture and gender roles.

#### The story of pain

The experience of pain manifested in many different forms. Some women experienced the demoralizing effects of chronic physical pain:

*“Since that time, till now I’ve got pain on my right hip, back and leg, the whole like all into my toe…it put me through a lot of things, like I can’t walk, I can’t sit for long, I can’t even in sleeping, I can’t sleep much because of the pain, like it’s it’s horrible, it’s really bad… but I am dealing with it… I took pain killers, it didn’t work, I used to wake up in the morning my head just wants to blow up, I couldn’t be bothered doing anything” (P6, aged 35 yrs)*.

This pain impacted their ability to engage in day-to-day life:

*“Yeah, it did actually, especially the shoulder pain, the shoulder pain, sometimes I was driving and it would hit me, and like I had this one [daughter] in the car and I’m like oh I can’t drive anymore, so I would have to stop aside and just you know rub my shoulder or do whatever it took to calm the pain down or take a tablet…” (P1, aged 27 yrs)*.

All participants expressed frustration and a sense of helplessness most days over their pain. The pain experience was unpleasant, and participants were often unable to perform their normal activities of daily living.

*“The pain, it makes me feel like angry, like when I can’t sometimes walk, I can’t get on my feet in makes me like angry, like frustrated” (P9, aged 58 yrs)*.

Sometimes the pain meant that the participants were not able to engage in regular employment, as one woman describes the challenges she faced with work because of her chronic shoulder pain:

*“This pain in my shoulder, when they look for me, for the work, she can’t look [for] anything because I can’t carry any heavy thing” (P2, aged 55 yrs)*.

Uniquely, some women felt that the suffering they had lived through was and continues to feel deeply painful and led them to describe their physical pain through an affective lens:

“*…it was very hard, yeah, so hard, very painful, even I moved here but I can’t forget it, it is still on my mind. So was like very scared, if you see it, you say why they do it like that. Maybe you see it on the news, sometimes, I think, in Iraq, they do bombs, like the war, so was in Northern Iraq. Thank God they didn’t come in Baghdad. We had Army people, they us. The Iraq Army, very helpful people, they save us… my pain is psychological too, so I was always like crying… even my dad, moved me to here, he was scared about me*…*like a, I was seeing so many people killed from my eyes, that was scary, that was pain” (P4, aged 18 yrs)*.

Almost half the women described feelings of inadequacy and guilt because they are in pain and because it challenged their role as caregivers and as mothers.

*“Yeah, that was my main concern, [caring for my daughter] because I just can’t do anything for her while I have got this pain.” (P1, aged 27 yrs)*.

##### The experience of help seeking in Australia and home country

There were a range of experiences the women expressed regarding the health care that they had received for their chronic pain. Overall, most expressed a sense of gratitude for Australia and the availability of healthcare at no cost, particularly when comparing to the availability of supports in their home country:

*“… everything is good, very good, I have been very happy with the care I have been given in Australia” (P9, aged 58 yrs)*.

Many also expressed this gratitude towards their regular GP, many of whom who had been seeing the same GP since first arriving to Australia:

*“ … he is really, really, really good doctor, I am so happy, everything, he is very good…he takes like, really extra care of us, and all the medication he prescribing it was working very well, and he always sends me places for example, it’s so good, I am so happy with him… yeah…this doctor is very good, he listens to me” (P9, aged 58 yrs)*.

In general, participants expressed that the level and conditions of care received in Australia were better than those in their home country:

*“Oh, very large difference… from here and Iraq, very large difference, yes… here is very, we get very good care here, in Iraq, we don’t… no, not much care, compared to here” (P10, aged 79 yrs)*.

This sense of appreciation was partly due to the stark differences in care received in their home country which would be expected considering the continued conflict that exposed them to violence, loss and trauma. The exposure to ongoing corruption, war and suffering made access to quality health care for chronic pain difficult. For example, one woman described visiting a family member in hospital and the poor conditions that her brother was receiving care in:

*“Ugh, I went to the hospital once to visit my brother, he was sick and it was disgusting… it was actually disgusting, and there was no power, no water, nothing, yeah it was just… horrible” (P6, aged 35 yrs)*.

#### Factors shaping the ability to engage with care

##### Language

There were a range of issues regarding language including interpreters, family, language of the treating doctor and confidentiality. Overall, participants shared that the inability to speak English made things challenging and were depended on members of family to be present during medical appointments to act as key informants.

*“The doctor, speak Arabic? No, my niece, she is my carer…in terms of accessing health care… yeah because of language it has been hard but thank God I have my niece, and if she is not able, her father or my sister they come with me…” (P9, aged 58 yrs)*.

Often, if communication and English language were unclear, women felt misunderstood, particularly when doctors used medical terminology. This led to inconsistencies among health care staff during medical and administrative procedures which made seeking care for chronic pain even more challenging. Participants were also often not able to clearly describe their diagnosis because of the medical terminology used during their appointments. This was also because many doctors did not provide interpreters or translated material during their consults:

*“It was difficult, like I can understand from the doctor but sometimes they say medical words, and I can’t understand it… like I can understand him, but when he says those medical words, it is difficult, yeah, I am still learning, all the health words… And when I see the surgeon, I keep telling him, sometimes I can’t understand your medical language, so I have to book interpreter, he says okay, doesn’t matter, if he understands or not, sometimes I try see if my uncles wife can come, so she can understand, and she can tell me what they say, when I can’t have interpreter” (P4, aged 18 yrs)*.

In contrast, some women placed significant trust and confidence in family members to interpret during medical appointments and thought that having a family member translate medical terminology over a professional interpreter was sufficient:

*“…he spoke English, my son come with me and he understand what he says and he explained it to me…no I don’t ask about interpreter, when I go with my son, I don’t ask” (P8, aged 80 yrs)*.

Having an Arabic speaking doctor was also important. Many felt that sharing similarities in cultural beliefs and behaviours which could be understood by their treating doctor was instrumental for providing effective communication during their consult:

*“Actually I need someone who understands me and what I need, that’s why i want to see an Arabic speaking GP” (P4, aged 18 yrs)*.

However, this was challenged by one woman who felt that seeing a doctor who was a member of the community meant that certain issues and examinations were not discussed due to judgement and concerns around confidentiality, as she describes:

“No, especially, the GP, is like a friend, not of the family but it is a friend of ours, so this why I don’t feel very comfortable, even for something for woman health so I prefer a female GP” *(P3 aged 45yrs)*.

##### Gender of provider

The women’s ability to engage with the health care system was influenced by the gender of the treating doctor. Some women reported feeling more comfortable discussing their pain and concerns, particularly if relating to women’s health, with female GPs and felt a sense of judgement or discomfort if the doctor was a man:

*“…I just feel like I can explain everything, uhm so I have got another GP for my children, he’s a male, and I’ve been to him, I’ve tried before and I just feel really uncomfortable speaking about private parts and how I’m feeling and I feel like he’s also a bit hesitant, uhm I don’t know if it’s because of his religion, I know he’s… I don’t want to speak about his religion but I know that he’s Muslim, and I feel like he’s uhm maybe because of that he’s too embarrassed to speak about it, and now so am I, and now so that why I only go to him if I’m like. sick or like I’ve got the flu or you know, like my arm hurts, but I wouldn’t go to him for like, uhm to like put a Mirena in, a contraception, or like uhm you know can you check my boobs if I have cancer or something,” (P7, aged 29 yrs)*.

##### Awareness of health systems

Overall, many of the women were not very trusting of some services. This showed striking insights into their knowledge around preventative care, the continuous and integrated nature of chronic pain management, and meant that many of the women did not want to engage in some health services despite no explicit gap of existing providers:

*“I think I was doing everything I could to manage it, because I thought, like who, like with the allied health with the Chinese medicine and stuff, actually I did look at massage, and not physio, but maybe massage could help calm down my pain but I never ended up going because I was like, I thought if all this medicine is not helping how is a massage going to help chronic stomach pain, yeah but yeah I just didn’t end up going…Cause I just thought, is it going to work or not…it was just that I didn’t think it would help” (P1, aged 27 yrs)*.

Some participants felt they received insufficient information on the services available and thus face difficulties navigating the health care system upon arrival to Australia. Particularly, women felt uninformed about the health issues with respect to gender and female-specific pains. As this 45-year-old woman suggested; she had felt a conflict between the information that she knew and the information she had been given:

*“I heard from my friends that they did this kind of test [cervical smear], but my GP didn’t tell me, maybe because he is a male or I don’t know, I don’t know if it is related, but this is also important. When a refugee woman, comes here, that they can give her this knowledge that you need to do this kind of women’s health test, and this kind of test, at this age and maybe give her a list of this of what she has to do every two years or every 5 years, you understand what I am talking about?… so I just heard from my friends, that I did something like this and you need to do it when you reach 40 yo or something of 45 yo, so I think this is important also to give this knowledge to the woman when she arrives here, so that may be important as well…yeah, so I think this is important when we arrive that we are given this kind of information around women’s health” (P3 aged 45yrs)*.

##### Affordability

Some women expressed that the inability to afford private health care services is a barrier for accessing specific chronic pain health care. Participants stated that financial difficulties were in part exacerbated by competing priorities such as family welfare as one woman described:

*“…I was like anything that can help, I don’t care I will get it, it was expensive, but then they didn’t end up prescribing it, it had to be prescribed, I could and I couldn’t afford it, but if it’s going to take my pain away, I don’t care I would get it, anything to help with this sort of pain, like it would break the budget but it wouldn’t keep me hungry” (P1, aged 27 yrs)*.

Financial difficulties also showed that some patients could not afford the money to receive the adequate care they needed.

*“At the time he told me if you want to do the operation you need fifteen thousand dollars from your own pocket to do the operation, or you can do health insurance for a year, and then you can come here and I will do it for you…I really want to do it, I can’t wait to do it. Because I couldn’t afford the money, that much money I couldn’t afford it. And uhm I wanted to do the health insurance and I called the actual surgeon to give me the item number, he didn’t, he didn’t give me the item number. So I can’t do the insurance until I have the item number” (P6, aged 35 yrs)*.

Additionally, there were several challenges for refugee women to navigate the prolonged wait-times to receive care in the public hospital system. This protracted period living with pain due to the long-wait times lead to ongoing daily challenges when it came to managing and living with their chronic pain:

*“He didn’t mention anything about it. He said we will do the papers, and we will send them to the Hospital and see what they can do. Maybe three years on the waiting list, because this kind of operation they don’t do it as much… six years I have been waiting in pain and I have five kids and uhm [Government agency] want me to work, this is the bad issue, they want me to find a job… my younger one turned six and I have to find a job” (P6, aged 35 yrs)*.

#### Support seeking systems – GPs and family

In Australia, GPs act as key support persons and trusted health professionals who are typically the first point of care for most patients. This was true of most the women’s experiences and expressed that GPs advocated for them and served as an important gateway for any further referral to other health or pain specialty services:

*“I’d probably go to get it checked out, I don’t know, who would I go to, probably my GP, I would just go to my GP, and they would recommend someone I guess? GP would be my first point of call” (P7 aged 29 yrs)*.

Participants felt a deep trust and connection to family, and often made health decisions based on their advice and recommendations. Although participants mentioned connecting with refugee health services upon arrival to Australia, many relied more on family and community members to navigate health care systems when it came to more complex health issues like their pain several years into their resettlement. Family members were typically responsible for introducing the women to local health services particularly when it came to first connecting with an Arabic speaking GP in Australia:

*“ He [GP] was very good … because he was my sister’s GP as well, so through them I know him, so I started going to see him” (P9, aged 58 yrs)*.

Family members were also fundamental in linking the women to other allied health services that were not recommended by their GP. This meant that much of the information refugee women received was based on the personal experiences of other family members and friends:

*“My mum tried the Chinese needles, acupuncture I think it is called this, this helped her I think, she has some pain…even my husband got an appointment with him. I think it helps.” (P3 aged 45yrs)*.

And making medical attendance was also heavily reliant on family members availability to take them:

“Actually, it is hard for me to get to the GP, my carer takes me, because he is like one hour and half drive, so my niece take me, but to me, the doctor is more important than location, I am willing to travel for a good GP” *(P9, aged 58 yrs)*.

Furthermore, family and social support networks were an integral aspect to coping with the pain. Many of the women relied heavily on the support of family members for emotional support.

*“You know it was more mental support, than physical, which is still good, rather than physical support, but any support is my family I rely on a lot, so like my mum” (P1, aged 27 yrs)*.

Family members were also key when it came to providing physical support with coping with pain and activities of daily living as described by one woman below:

*“My husband he was doing the massage for me, always, very good to me, he would help me in the shower too, before the surgery and even after the surgery. Before the operation and after, very good my husband, my husband 26 years we are married, I love my husband very much” (P5, aged 48yrs)*.

#### Influence of culture and gender roles

Culture and gender roles shaped many of the ways in which the women managed their pain. Refugee women arriving in Australia are confronted with social, cultural and environmental conditions that are very different to their country of origin. The Assyrian culture has long faced ethnic and religious persecution, and many have fled their home countries as a result, in hopes of a better life:

*“Ah, well, it’s not easy, to move to another country, from culture to another culture, from language to another, so lots of things that’s different, so to settle down in Australia… we, needed sometimes to do this, my focus was to let the family settle, especially the kids, to settle in their school, to and we needed sometime to know Australia and the how the life is here and the focus was for my husband to get a job, kids go to their school…” (P3 aged 45yrs)*.

Furthermore, Assyrian women are often expected to adhere to gendered specific roles rooted in family welfare and household duties that at times serves to diminish their own agency. These cultural values influenced their own social identities, perceptions and advocacy around health decisions, and as a result held significant influence on shaping and forming the models of thought around life and their struggles with pain. As one woman describes:

*“He [husband] say, Linda you should be responsible for them, oh for everything, make sure they finish their study, and I have nobody no another family, from my side. Sister, brother, for me, nobody here” (P2, aged 55 yrs)*.

Competing priorities oftentimes as mothers and caregivers meant that many women did not have the flexibility to wait several hours or drive long distances to seek care that supported good communication:

*“It’s very difficult, I don’t know anyone that speaks Arabic at the moment, like any GP, unless, besides from the one when we first came to Australia, she’s still there, she still exists, but she’s sooo busy, she’s soo busy… like to get into her clinic you need like three hours to wait, you know that’s not something I want to do… uhm my kids would be left alone for three hours, I can’t do that, I’d have to take them with me, and over there that would be a big struggle. you know… yeah” (P7, aged 29 yrs)*.

Despite acknowledging that life in Australia, and expectations for women are different, there are still patriarchal and cultural constraints for some refugee women trying to access care relating to pain:

*“… maybe I don’t want to be like a weak woman, so he (husband) will know that he controls me even more. Anyway I think he is better now than before. Maybe the life here is different, because maybe he feels that woman here have more rights, so he is better than before but there is still, he do some kind of behaviours that is not acceptable.” (P3 aged 45yrs)*.

One woman giving the example that she feels her husband controls her decisions, even those around her health.

*“Even if he is not in the house, he told me that, I am not allowed to leave house, he needs to know when I leave house, when I will go and maybe how long I will be so yeah he wants to control everything…mm yeah, he like to control me.” (P3 aged 45yrs)*.

Some women also described a sense of shame and stigma around seeking care for the psychological aspect of their pain. Part of this shame came from family members and the community. Cultural differences made seeking care for mental health difficult and often presented as a barrier as one woman described:

*“No psychologist, because it is also not common, in our culture to do this… we don’t do something because it’s not acceptable in our culture… I would see a psychologist if I didn’t feel judgement” (P3 aged 45yrs)*.

One participant suggested that if mental health care plans were made available and were a routine requirement for all refugee women upon arrival to Australia there would be less judgement from others:

*“There is judgment although lots of people need to do this, go to psychologist, because of what has happened to us before and how we survived and came here and lots of things affect, so this something important I think should be involved…So I think it is important to involve psychotherapist from the beginning when people arrive here, as what happens is there are some, what’s called, institutions which is to take care of refugees when they arrive, So when we arrived, the person from them came to us to take us to Centrelink, to the bank to open up an account, to help to enroll to enroll in school for kids and for us, all this…so I think in this time, it’s very important to involve psychotherapist with these things…it’s like, ah, process for everyone, more accepted. And later, the specialist [psychologist] should also follow up with people for a while… yes this psychologist, for a while with people until they can settle down here and feel okay for them, and they don’t need more psychologist help” (P3 aged 45yrs)*.

## Discussion

This phenomenological study provides novel and unique insights into the experiences of Assyrian refugee women’s seeking care for chronic pain several years into adjusting to new life in Australia. Our study identified five major themes arising from participant interviews.

Firstly, the story of pain for refugee women is unique and fraught with adversity, and the experience of health seeking in Australia is different to the participants’ home countries. Furthermore, refugee women’s engagement with health care services was influenced by factors such as language, affordability and the gender of the provider. Key advocates such as GPs or community and family members were also essential in facilitating this access to chronic pain services. Lastly, our study demonstrated that family welfare was a central component to access and highlighted the influence of culture and the impact gender roles placed on refugee women’s willingness to seek help for their pain.

These findings are consistent with the Levesque’s et al. (2013) Patient-centered Access to Health care framework which defines access as the opportunity to reach and obtain health care services in situations of perceived need for care [9]. Exploring access from the perspectives of refugee women shows the unique interface between the individual characteristics of the women, their households, social and physical environments and the characteristics of health systems, organisations and health care providers [12]. Factors to consider therefore also pertain to the features of populations, and the ways in which access is realized in women of refugee background living with chronic pain [9].

In line with Levesque’s framework, our research shows that the ability for women to engage with health care services were underpinned by empowerment struggles, health literacy, adherence and caregiver support. There were also challenges regarding their ability to pay and reach services particularly with regard to referral-based care such as specialist pain clinics and allied health care. Many women were able to access primary care physicians through family, community networks or resettlement service providers; however, their ability to reach specific pain management services were met with several challenges. As Levesque’s framework suggests, the ability to reach specialist services was in part due to their living environments, their transport and social support networks. Furthermore income, assets and health insurance knowledge also impacted their ability to engage with health services beyond primary care. There are also factors that may influence whether people, particularly those who intersect with multiple vulnerabilities accept a service based on pre-existing beliefs and experiences [29, 30]. Women’s gendered experience during war and civil conflict, combined with the stressors they encounter in exile make their needs comparably unique to those of men [15, 31] and may impact how they perceive health care provision, their pain and the appropriateness for them to seek care.

When considering access to health care, our findings suggest that the appropriateness or the ‘fit’ between refugee women’s needs and the service, its timeliness, the care spent in assessing their complex health needs and determining the correct management, is essential. Our study has shown that refugee women and their ability to engage in health care can be challenging. Levesque and colleagues (2103) describe the ability to engage as relating to the participation and involvement of the client in decision-making treatment, which is strongly determined by the capacity and motivation to participate in care. However, refugee women’s engagement is related to their capacity to communicate as well as be health literate, have the willingness to advocate for themselves and be willing to receive appropriate care [32]. In line with our findings, Floyd and Sakellariou (2017) demonstrate how fractured sovereignty can add to the layers of disadvantage refugee women experience, particularly with regard to compromised choice and a lack of autonomy in health care decisions. In particular, Floyd and Sakellariou (2017) illustrate how gender and refugee status are not discrete categories and highlight the different ways vectors of disadvantage intersect with each other to produce the possibilities for exclusion or compromised access and autonomy for refugee women seeking care.

Participants reliance on family members to assist with interpreting during medical appointments may, in part, be due to a lack of trust towards interpreting services. However, professional medical interpreters offer an essential role in health communication particularly when describing the complex management involved with chronic pain, which has shown to improve patient experience and quality of care [33–35]. Some participants also sought out doctors from the same community so that they could speak freely in their own language. Interestingly, women from our study perceived this differently as privacy and cultural sensitivity concerns came into question. Autonomy is largely influenced both by culture and more systemic barriers such as English language fluency during medical consults and engagement with health care systems [36, 37]. Safe and optimal healthcare relies on the clear exchange of information between health care providers and people accessing their services. However, language fluency particularly in medicine is often a major obstacle for refugee women trying to receive equitable and high-quality care in host nations [38].

The women in our study expressed a range of opinions regarding the gender preference of their health care provider. Many women did not express gender preference so long as they were satisfied with the care they were provided. Whilst gender may be one aspect of the professional providing care, this characteristic may have a greater influence both on the access and outcome of care. Based on the intimate nature within consulting rooms, the gender of the health care provider featured prominently in the narratives of some Assyrian refugee women interviewed. A study by Ahmad and Colleagues (2002) found that the majority of Cambodian immigrant women demonstrated similar gender preference patterns when choosing a family physician [39]. The same preference was also noted when deciding on the use of a medical interpreter, which has been shown to facilitate better communication for refugee women with limited language proficiency [40, 41]. Possible explanations for this preference include the level of clinical care [42–44]; physician’s attitude, communication and practice style [45, 46]; preconceived stereotypes [47]; the quality and nature of past experiences [42] and age of the patient [48]. Cultural beliefs, norms and attitudes affect the manner in which health, pain, and source of treatment are perceived [49], particularly in Western countries such as Australia where a significant population is comprised of refugee and immigrant women.

Women of refugee background also face challenges that are inherent in negotiating their new roles in resettlement. In particular, there is a prominent theme of family welfare, which is typical across many cultures and dates back to premigration history of culture and family dynamics. A study by Chung and Bemak (1998) explored the lifestyle of Vietnamese refugee women and demonstrated that people are seen within the family structure rather than individuals. Their study revealed that women are primarily expected to show obedience to their husband and serve and care for their children [50]. Patriarchal paradigms exist across many cultures often demanding that women remain dutiful to their family, often at the cost of their own health and well-being [50]. However, agency should be considered within particular contexts [51]. For refugee women, agency is embedded in both family roles and a sense of community belonging [52]. Kanal and colleagues (2021) suggest that studies of agency in refugee and migrant women would benefit from identifying culturally grounded stressors and coping strategies according to the specific context and avoid viewing women as passive victims. Interestingly, Kanal and colleagues (2021) argue that agency can also be seen in refugee women’s everyday attempts to mother and make new homes and to re-evaluate how we interpret the ‘sacrifices’ made by refugee mothers for the sake of their children and family well-being. Actions that may be typically interpreted as detrimental to their health should consider the moral injury that may ensue by the inability to fulfill a mother’s role. In doing so we can understand the positive effect on their subjective well-being and also the agency enacted through this way of coping [52].

Refugee women often arrive at their host country with pre-existing health problems, particularly around trauma and pain that are related to their experiences of war and conflict. Hence chronic pain and related functional disabilities are highly prevalent among refugee women, which is in part due to a system that favors a male-centered paradigm [16]. There is a need to encourage refugee women’s agency over their own health care decisions and further explore how the influence of gender, low literacy and refugee status impacts women’s access to care. This, in part, could be enhanced through targeted and purposeful education implemented at a scale by professional organisations, primary health care networks and through the introduction of complimentary system level changes that encourage community engagement. In particular, using cultural community and primary care networks to enhance access to referral-based services such as chronic pain clinics and allied health services. Furthermore, studies of women empowerment and agency could benefit from identifying stressors and coping strategies according to specific cultural contexts. By attending to cultural contexts, migration researchers can avoid the mislabelling of women from refugee backgrounds as passive victims and may work towards developing more effective ways to support their everyday struggles with chronic pain and engagement with health care services. Primary health care may have a significant role in shaping these economic and social development programmes to advocate for knowledge-building interventions and work towards anti-oppressive practices, policies and research by considering refugee women’s experiences around health care in their host country.

There are some limitations of this study that should be noted when considering the findings. The qualitative nature of the study relies heavily on self-reported data which brings into consideration certain limitations. The number of participants included in the study also limits the generalisability of the study findings. The use of interpreters often resulted in fragmented conversation with some participants [53, 54]. Some participants also preferred to use family members as interpreters during their interview, which meant that some women may have been hesitant to discuss their experience in great detail [55]. In addition to this, some interviews also took place in public spaces, such as libraries or in the family home where other members were nearby. This meant that whilst confidentially was maintained, given the personal reflections often discussed in association with pain, trauma, and mental health some of the women may not have felt comfortable to fully express their opinions. Where possible, family members were asked to leave the room for the duration of the interview. Lastly, participants level of acculturation may have influenced the study findings through social desirability bias or the misrepresentation of cultural practices. However, to ensure we included diverse perspectives and experiences in our sample, the research team regularly engaged in reflexivity to critically examine their own cultural assumptions and biases.

### Conclusion

This study provides rich insights into the experiences of Assyrian refugee women living with chronic pain. Refugee women arrive in Australia with a host of needs and as such, it is important to include seldom heard, hard to reach population’s perspectives and facilitate their participation in research to understand how vectors of disadvantage intersect. Our findings suggest that affordability, language fluency, and health system literacy can influence access to care. Competing priorities such as family welfare may also lead to medical neglect, a cultural phenomenon unique to many refugee women. For successful integration into health care systems of host countries, particularly for complex conditions such as chronic pain, there is a need to work with women community members to develop programs that support informed decision making and ease of access health care pathways. This study provides the foundations for ongoing work to build programs centered around cultural practices that foster resilience and autonomy, particularly in primary care settings. This may be facilitated through settlement services alongside interpreters [56] and the implementation of a bottom-up approach, whereby refugee women could develop strategies that might contribute to reducing current health inequities.

## Electronic supplementary material

Below is the link to the electronic supplementary material.

Supplementary Material 1: Interview guide.

## Data Availability

Transcripts from the interviews are confidential and not publicly available. A subset of de-identified data are available from the corresponding author.

## List of abbreviations

GP: General practitioner
