# Supplementary material for: Experiences of Assyrian refugee women seeking care for chronic pain: a qualitative study
Source: Int J Equity Health. 2023 May 8;22:83. doi: 10.1186/s12939-023-01891-w (PMC10169379; doi:10.1186/s12939-023-01891-w)
Supplement: Supplementary file 1 — Supplementary Material 1: Interview guide. [file 12939_2023_1891_MOESM1_ESM.docx]

**
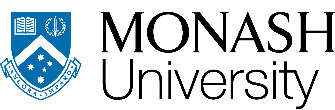
**

**Interview Guide**

**29/06/2021**

Hello, my name is Areni, I am a PhD student at Monash University and a clinical Osteopath. Thank you for agreeing to participate in this interview.

The aim of this chat is to gain a better understanding of your experiences navigating the Australian health care system to receive care for your chronic pain. I would love to know a bit about your background and some of the health and social support services you have access to, to understand how we can improve the way we provide care for refugee women living with chronic pain.

The questions in this interview should only take around 60 minutes, there are no right or wrong answers to any of our questions, I am just interested in your own experiences. Feel free to let me know if you don’t understand any of the questions and don’t feel like you have to answer everything.

**Before we start this interview, I just wanted to remind you that**

- *Your choice will not affect how you will be treated in the medical system*
- *All information will remain private, no one will know who you are, as no names are ever reported*

**May I turn on the digital recorder?**

**Background**

1. Can you please tell me a bit about yourself?

- ***Prompts:*** Gather information on country of origin and region, where were you born? How long have you lived in Australia?
- ***Prompts:*** Do you work? What do you do for work?
- ***Prompts:*** Are you married? Do you have kids at home?

**Impact of pain on everyday life**

1. In general, how is your health?
2. Is your chronic pain impacting your daily life?

- ***Prompts:*** what activities? Is it impacting your work and social life?

1. How do you manage every-day life?

- ***Prompts:*** What are some of your coping mechanisms? Do you mediate, exercise etc.?
- ***Prompts:*** *Do you have social support, through friends or family?*

1. What are three activities that are important to do, that you find difficult to do because of your chronic pain?

- ***Prompts:*** I.e. cooking, cleaning, driving?
- ***Prompts:*** How well can you perform those activities?

**Chronic pain and seeking care**

1. Can you tell me a bit about your chronic pain, when it started to now?

- ***Prompts:*** When did it first happen? What made it worse or better? Did you have it prior to coming to Australia?
- ***Prompts:*** What are you doing to manage your pain? Who did you turn to for help/advice? Do you see a specific health practitioner for your chronic pain? Have there been any changes to the way you understand your condition?
- ***Prompts:*** Tell me about that visit. How was the communication during the consult? How many times did you visit the service? Have there been any changes to the way you understand your condition? What would you change?
- ***Prompts:*** Were you provided any resources that assisted you in understanding your condition? (e.g. pamphlets, pictures, etc.)? If so, how useful were they?
- ***Prompts:*** Was it difficult to find treatment for your chronic pain?
- ***Prompts:*** Are you satisfied with it? What aspects of your visit do you like and dislike? Is it what you had hoped?

1. If care has not been sought: Why not? What kind of care would you want to receive?

- ***Prompts:*** What kind of care would you want to receive? What are the most important results you would want from receiving such care?

1. Are there any services that you would like access to?
2. In your opinion, what are some of the important aspects of care that you would like to receive?

- ***Prompts:*** Would you like better communication? More knowledge of services?

1. If you don’t mind me asking, were there any financial barriers to receiving care?

- ***Prompts:*** Were there any other barriers?

**Conclusion**

1. Is there anything else you would like to tell me that I haven’t already asked you about?
